# Design and Analysis of a Fluid-Filled RF MEMS Switch

**DOI:** 10.3390/s23052692

**Published:** 2023-03-01

**Authors:** Hongyu Zhu, Wenhao Cui, Yanzhang Li, Mingxin Song

**Affiliations:** School of Applied Science and Technology, Hainan University, Haikou 570228, China

**Keywords:** radio frequency (RF), MEMS, insertion loss, driving voltage, impact velocity

## Abstract

In the present study, a fluid-filled RF MEMS (Radio Frequency Micro-Electro-Mechanical Systems) switch is proposed and designed. In the analysis of the operating principle of the proposed switch, air, water, glycerol and silicone oil were adopted as filling dielectric to simulate and research the influence of the insulating liquid on the drive voltage, impact velocity, response time, and switching capacity of the RF MEMS switch. The results show that by filling the switch with insulating liquid, the driving voltage can be effectively reduced, while the impact velocity of the upper plate to the lower plate is also reduced. The high dielectric constant of the filling medium leads to a lower switching capacitance ratio, which affects the performance of the switch to some extent. By comparing the threshold voltage, impact velocity, capacitance ratio, and insertion loss of the switch filled with different media with the filling media of air, water, glycerol, and silicone oil, silicone oil was finally selected as the liquid filling medium for the switch. The results show that the threshold voltage is 26.55 V after filling with silicone oil, which is 43% lower under the same air-encapsulated switching conditions. When the trigger voltage is 30.02 V, the response time is 10.12 μs and the impact speed is only 0.35 m/s. The frequency 0–20 GHz switch works well, and the insertion loss is 0.84 dB. To a certain extent, it provides a reference value for the fabrication of RF MEMS switches.

## 1. Introduction

Devices fabricated using RF MEMS technology have been widely used in defense and medical fields because of their low power consumption, high integration, high linearity, good isolation [1,2,3,4], and wide frequency band. Due to the wide application in airborne and satellite-based radars and IoT communication systems [5,6], RF MEMS switches have become an important research hotspot in recent years. Early research focused on low driving voltage and microwave power, but as research progressed, it was found that RF MEMS switches have a long response time and are prone to mechanical breakdown.

By experimental demonstration, Goldsmith and others [7] demonstrated that the switch life can be extended by more than 10 years if the driving voltage is reduced by 5–7 V. Previously, Sakata [8] developed a high-reliability switch based on single-crystal silicon film using SOI silicon chip and substrate transfer technology. The glass fusion process allows the switch to be sealed and packaged. Although the driving voltage of the switch was reduced to some extent, the larger switch size resulted in a slower switching speed. The sacrificial layer material is Al, and the sacrificial layer structure is prepared by the release method and supercritical drying method, which can completely and effectively reduce the threshold voltage of the switch. However, due to the low stiffness of the support, mechanical breakdown is easy to occur after repeated use.

Previously, Ke et al. [9] identified that the dielectric in the gap was compressed and pressed onto the substrate from below, which could lead to severe damping, decreased switching speed, and reduced mechanical sensitivity. In 2021, Sravani [10] investigated the effect of film flatness and roughness on switching capacity and determined the best method to reduce redox. Presently, although the switching voltage can be reduced by optimizing the internal structure of the switch to reduce the spring coefficient, this reduction is often limited by reliability and feasibility.

Riverola et al. [11] designed a zero-stage vacuum packaged RF MEMS switch and achieved a switching time of only 77.5 ns at a drive voltage of 36 V. Initial reliability tests were also performed for 1700 switching cycles, and the results showed that the performance of the switch was not compromised after packaging. Zhen et al. [12] designed RF MEMS switches encapsulated in dry air and humid environments, and the switches made of silicon dioxide were less susceptible to surface charging than those made of silicon nitride at the same electric field and humidity levels, verifying new techniques to evaluate the effectiveness of dielectric preparation and encapsulation. According to Equation (8), the insulating liquid-filled RF MEMS switch can reduce the driving voltage of the switch, which slows down the falling speed of the switch, reduces the impact between the cantilever beam and the dielectric layer to a certain extent, and improves the service life of the switch. Therefore, a double-ended fixed capacitive RF MEMS switch is proposed, different filling media are used to simulate the internal environment of the switch, and finally silicone oil is chosen as the internal environment of the switch to analyze the effect of the change of opening voltage and falling time on the switch life, and provide a theoretical basis for the optimization of the RF MEMS switch.

## 2. Analysis of the Structure and Working Principle of the Switch

**Figure 1:**
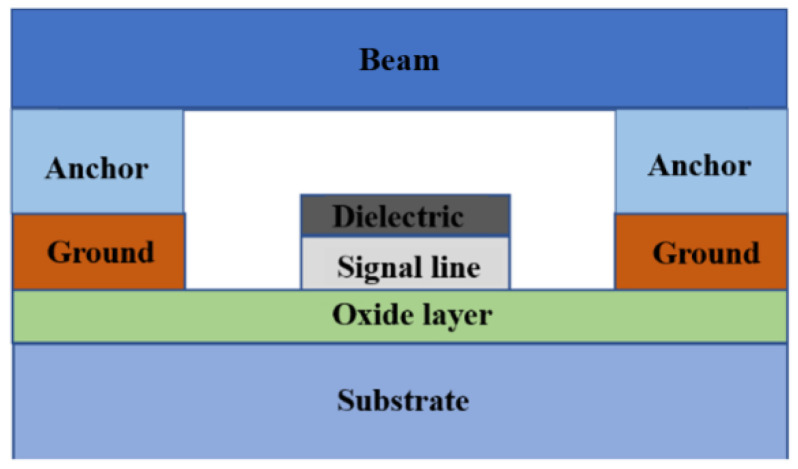
Cross-section of the RF MEMS capacitive shunt switch.

Regarding the dimensions of the designed switch, the length is 300 μm, the width is 60 μm, the height is 2 μm, and the coplanar waveguide (CPW) is the ground and signal line. The cross section and three-dimensional view of the RF MEMS switch is shown in Figure 1. It can be observed that the crossbar is located between two anchor points, and the two anchor points are set on both sides of the ground wire.

**Figure 2:**
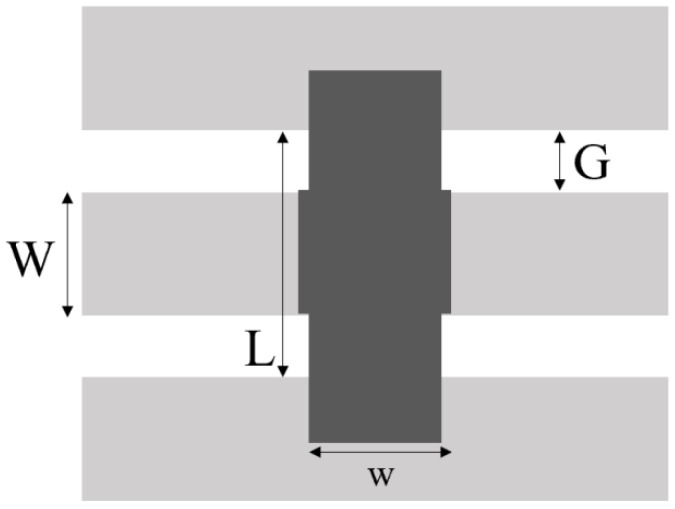
3D view of the RF MEMS capacitive shunt switch.

As can be observed in Figure 1, the bridge switch is modelled and fabricated using a conventional layout, i.e., an anchored beam bridge capacitor shunt switch, and the signal dielectric uses silicon nitride as the thin film material. In this experiment, silicon (Si) is used as the substrate, silicon nitride as the dielectric layer, and gold as the signal conduction material. The intermediate three-dimensional structure of the capacitance-switched RF MEMS is shown in Figure 2.

Figure 2 consists of several important parameters: L denotes the length of the cantilever beam, w denotes the width of the cantilever beam, W denotes the width of the center coplanar guide, and G denotes the distance between the center coplanar guide and the ground. The cantilever beam is connected to the coplanar waveguide ground through the anchor point, and the connection between the cantilever beam and the center guide corresponds to capacitor with normal signal transmission. When the driving voltage is applied between the cantilever beam and the central guide strip, the cantilever beam is pulled down under the action of the electrostatic force, and finally comes into contact with the insulating dielectric layer after being pulled to a certain height. At this time, the equivalent capacitance value between the cantilever beam and the central guide strip increases sharply, and the microwave signal is coupled to the ground through the capacitance, and the signal is reflected back to the input end, so that the signal is turned on and off.

### 2.1. The Working Process of Capacitive RF MEMS Switch

The motion of the sensitive structure is strongly affected by the fluid viscosity so the influence of the fluid viscosity on the switch needs to be considered after filling the medium into the RF MEMS switch, where a control equation for the incompressible fluid viscosity model is introduced as:

(1) $$\frac{\partial u_{i}}{\partial x_{i}}=0$$

(2) $$\frac{\partial u_{i}}{\partial t}+u_{j}\frac{\partial u_{i}}{\partial x_{j}}=-\frac{1}{\rho }\frac{\partial p_{rgh}}{\partial x_{i}}+v\frac{\partial }{\partial x_{i}}\left(\frac{\partial u_{i}}{\partial x_{j}}\right)-\frac{g}{\rho }\left(x_{2}-x_{r}\right)\nabla \rho$$

In the above equation, $u_{i}$ and $x_{i}$, respectively, represent the velocity component and the right-angle coordinate system component along the i direction, $p_{rgh}$ represents the pressure value of the fluid, $p_{rgh}=p-\rho g(x_{2}-x_{1})$, where g is the gravitational acceleration, $x_{r}$ represents the initial height of the filled fluid, ρ and v represent the fluid density and the kinematic viscosity coefficient, respectively.

A VOF method is proposed in the paper by Hirt and Nichols [13] to calculate the effect of fluid on the RF MEMS switch. In VOF, the motion of the fluid should follow the following equation:

(3) $$\frac{\partial \phi }{\partial t}+\nabla \cdot (av)+\nabla \cdot \left(a\left(1-a\right)v_{r}\right)=0$$

In the above equation, $v_{r}$ represents the velocity of the fluid flow, and the third term of Equation (3) is a virtual compression term introduced into the numerical solution process to improve the numerical dissipation of the free pages. ϕ represents the surface tension of the liquid, a represents the volume of the liquid filling switch, and a value of one means full filling. Since this switch is fully filled, the value a is set to one during simulation.

In this paper, the whole operating process of the shunt capacitive RF MEMS switch is divided into four parts:

(1) The initial state of the switch (up state), in which no driving voltage is applied, the switch is equivalent to two small capacitors in series, as shown below:
  (4) $$\frac{1}{C_{up}}=\frac{g_{0}+t_{d}/\varepsilon_{r}}{\varepsilon_{0}\varepsilon_{l}wW}+\frac{1}{C_{f}}$$
  In contrast to the classical capacitance equation, $\varepsilon_{l}$ is added to calculate the capacitance, making it easier to compare the results of the simulation with the actual calculation afterwards. Where, $\varepsilon_{0}$, $\varepsilon_{r}$, and $\varepsilon_{l}$ are the dielectric constants of the air, the insulating dielectric layer, and the filling medium, respectively; w is the width of the cantilever beam; W is the width of the central conduction strip; $g_{0}$ is the initial distance between the cantilever beam and the insulating dielectric layer; $t_{d}$ is the thickness of the insulating dielectric layer; $\varepsilon_{r}$ is the dielectric constant of the insulating dielectric layer. $C_{f}$ is the edge capacitance, which usually accounts for 20–60% of the capacitance value of the parallel plate. In the up state, the edge capacitance cannot be ignored. Because $C_{u}{}_{p}$ is small, the signal of the central guide band can be transmitted almost without attenuation during the transmission process.
(2) When a certain driving voltage is applied to the cantilever beam, the switch is in a pull-down state, and the equivalent capacitance $C_{m}$ between the insulating dielectric layer and cantilever beam increases rapidly, as the same time, it is necessary to consider the effect of filling medium on capacitance, so the following is introduced:
  (5) $$C_{m}=\frac{\varepsilon_{0}\varepsilon_{l}wW}{\left(g_{0}-g\right)+t_{d}/\varepsilon_{r}}$$
  where g represents the distance that the cantilever moves towards the insulating dielectric layer, during which the equivalent capacitance increases rapidly in value.
(3) When the switch is pulled down to come in contact with the insulating dielectric layer (down state), the equivalent capacitance of the cantilever beam and the insulating dielectric layer reaches the maximum $C_{down}$ in this case, it can be equivalent to a large capacitance. Considering the influence of insulating medium on capacitors, the following formula is defined:
  (6) $$C_{down}=\frac{\varepsilon_{0}\varepsilon_{l}wW}{t_{d}}$$
  At this point, $C_{down}$ corresponds to a large capacitor with respect to $C_{u}{}_{p}$, through which the signal is coupled to ground.
(4) After the driving voltage is removed, the electrostatic force on the cantilever beam is reduced, and the switch returns is restored to up state through mechanical restoring force. At this point, the mechanical force on the switch is much larger than the viscous force of the insulating medium, so its effect can be ignored.

### 2.2. Calculation of RF MEMS Switch Pull-Down Voltage

Where $g_{1}$ is the distance between the cantilever beam and the electrode, and the thickness of the dielectric layer is ignored.

In the calculation of the electric field force, the structure of the cantilever plate and the lower tension plate is simplified into a parallel plate capacitance model, the boundary effect was ignored, the influence of the insulating medium on the electrostatic force of the cantilever beam is considered, and the relative electrostatic constant of the insulating medium is introduced to verify the magnitude of the electrostatic force obtained from the subsequent simulation, so that the electrostatic force $F_{e}$ on the cantilever beam can be written as:

(7) $$F_{e}=-\frac{1}{2}\frac{\varepsilon_{0}\varepsilon_{l}wWV^{2}}{g_{1}}$$

where $g_{1}$ is the distance between the cantilever beam and the electrode, and the thickness of the dielectric layer is ignored. At the same time, considering the influence of insulating medium on the pull-down voltage, the pull-down $V_{p}$ voltage is defined as follows:

(8) $$V_{p}=\sqrt{\frac{8k}{27\varepsilon_{0}\varepsilon_{l}wW}g_{0}{}^{2}}$$

where k represents the elastic coefficient of the cantilever beam, and k can be calculated by the following formula:

(9) $$k=32Ew{\left(\frac{t}{l}\right)}^{3}\frac{1}{8{\left(x/l\right)}^{3}-20{\left(x/l\right)}^{2}+14\left(x/l\right)-1}$$

where E is the modulus of elasticity of the cantilever beam material, w is the width of the cantilever beam, t is the thickness of the switch, and l is the length of the cantilever beam. When the load is applied uniformly to one-third of the length of the center of the fixed cantilever beam, the coefficient of elasticity of the turnout can be calculated as follows:

(10) $$k=32Ew{\left(\frac{t}{l}\right)}^{3}\times \frac{27}{49}$$

This means that when the cantilever beam is down to one-third of the way down, the cantilever beam can be made to attach to the dielectric layer by continuing to increase the drive voltage by a small amount.

### 2.3. Calculation of RF MEMS Switching Time

The switching time of the parallel capacitive RF MEMS switch consists of two sections. The first section is the time required for the cantilever beam to move from the initial position to contact with the insulating dielectric layer, and the second section is the time required for the cantilever beam to recover from contact with the insulating dielectric layer. Due to the addition of an insulating medium, the influence of the insulating medium on the drop time of the switch should also be considered at this point. The operating time of a switch is closely related to the switch’s construction, material properties, and operating environment, so the switching time is usually determined using simulation software. A short switching time results in a faster switching frequency.

### 2.4. Capacitance Ratio of RF MEMS Switch

The ratio between the down-state capacitance and the up-state capacitance of the parallel capacitor RF MEMS switch is called the capacitance ratio, as shown below:

(11) $$\frac{C_{down}}{C_{up}}=\frac{\varepsilon_{0}\varepsilon_{r}wW}{t_{d}}(\frac{g_{0}+t_{d}/\varepsilon_{r}}{\varepsilon_{0}\varepsilon_{l}wW}+\frac{1}{C_{f}})$$

The capacitance ratio of a switch is a very important performance indicator that represents the performance of the switch. The ideal capacitor ratio is infinite. In practical terms, the larger the capacitor ratio is, the more preferable it is.

### 2.5. Loss of RF MEMS Switch

Under ideal conditions, the RF MEMS switch can pass through the transmission line without loss in the up condition, but the addition of the filling medium will lead to a small equivalent capacitance in the up condition, so there will still be signals coupled to the ground through the capacitance and cause insertion loss and thus bring about signal attenuation. Here we reduce the thickness of the insulating medium layer to reduce the capacitance in the up condition and thus reduce the insertion loss, and subsequently through finite element physics, the insertion loss of the switch is obtained by subsequent finite element physics field simulation. When the cantilever beam is in the up state, the ratio between the output power $P_{out}$ and the input $P_{in}$ power is then taken as a logarithm, denoted by $S_{21}$, and is defined as follows:

(12) $$S_{21}^{up}=10\mathrm{lg}(\frac{P_{out}}{P_{in}})$$

After adding liquid to fill the RF MEMS switch, $P_{out}$ and $P_{in}$ are equal in the ideal state. The actual situation of insulating liquid will affect the presence of up-state capacitance; insertion loss is not 0.

### 2.6. Isolation of RF MEMS Switch

Switch isolation in the ideal state the greater the better, due to the addition of liquid-filled RF MEMS switches in the down state which will affect the isolation of the switch, specifically defined as:

(13) $$S_{21}^{down}=10\mathrm{lg}(\frac{P_{out}}{P_{in}})$$

Under the ideal state, the output power $P_{out}$ of the switch in the down state is 0, so the isolation degree is larger, and the isolation degree will not reach infinity because the capacitance of the down state is limited after adding insulating liquid to fill.

A new method to reduce the threshold voltage of a RF MEMS switch was investigated by exploiting the high dielectric properties of insulating liquids. The viscosity of the insulating liquid is used to reduce the shock velocity of the RF MEMS switch, and to avoid the negative effects of ambient humidity on performance of the RF MEMS switch. Meanwhile, reduction of the threshold voltage can extend the life of the switch and improve the stability and lifetime of the switch.

## 3. Manufacturing Method of Switch

**Figure 3:**
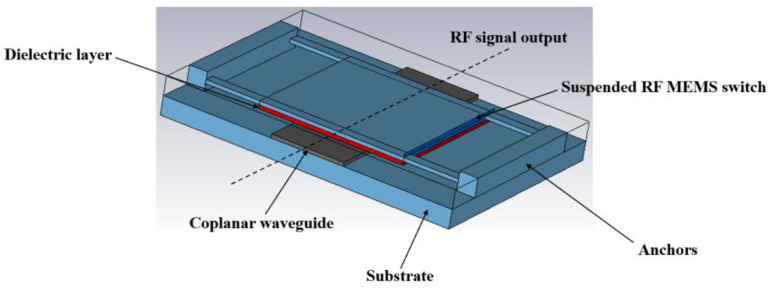
3D schematic of the RF-MEMS capacitive switch.

The 3D schematic diagram of RF-MEMS capacitor switch is shown in Figure 3. The insulating liquid-filled RF MEMS switch is prepared using the MEMS surface micromachining process, taking into account the requirements of the signal to the base in order to minimize loss and current leakage, using high resistance silicon as the substrate. 

**Figure 4:**
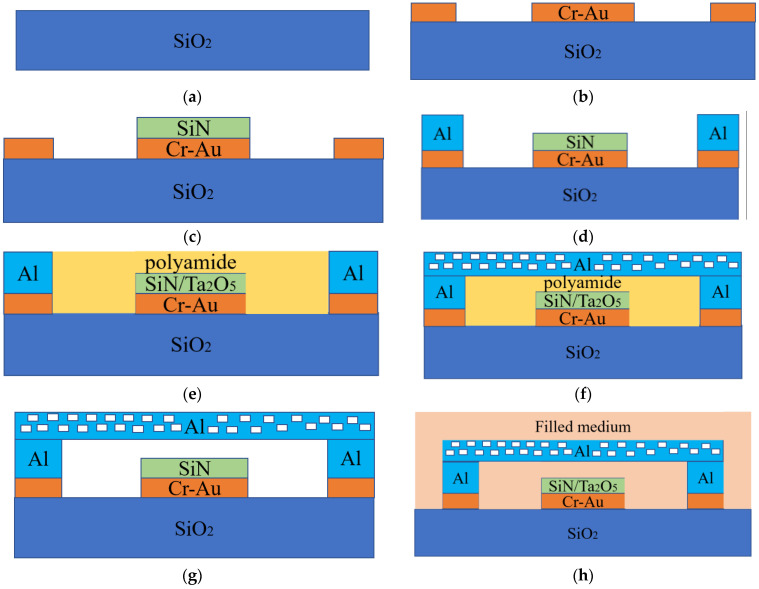

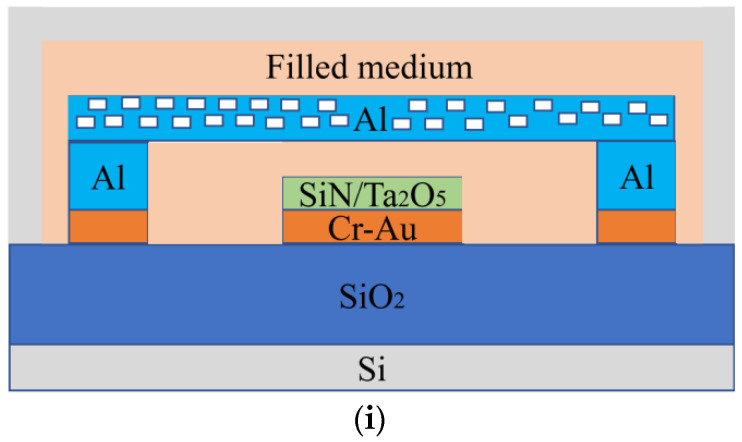
RF MEMS switch preparation process.

The preparation process of the switch is divided into 9 steps, as shown in Figure 4.

Step 1: Select silicon with high resistivity as the substrate material and form an oxide layer on the substrate surface by dry-wet-dry thermal oxidation process to make the substrate have good electrical insulation and prevent current leakage. See Figure 4a below.
Step 2: Forming signal lines and bottom electrodes of Cr-Au alloy by RF sputtering with positive adhesive stripping. See Figure 4b below.
Step 3: Sputtering a layer of SiN bilayer dielectric film, covering the CPW central signal line as an insulating layer, the thickness is 150/50 nm. See Figure 4c below.
Step 4: Evaporation of 3 μm thick AI, wet corrosion to obtain the support column. See Figure 4d below.
Step 5: Rotational coating of a layer of polyamide with a thickness of 3 μm, after lithography to obtain a sacrificial layer. See Figure 4e below.
Step6: Electron beam evaporation of a layer of Al for the upper electrode, wet corrosion out of the sacrificial layer release hole (small hole size of 8 × 8 μm). See Figure 4f below.
Step 7: Reactive ion etching to release polyamide sacrificial layer. See Figure 4g below.
Step 8: Standard supercritical dry ice treatment was used and filling medium is added. See Figure 4h below.
Step 9: Finally, a package is formed on a silicon wafer. See Figure 4i below.

To fill the RF switch MEMS with insulating fluid, silicone oil was finally selected through comparative analysis of different insulating fluids. Among the existing cantilever materials, such as gold and aluminum, considering the modernization of this experimental technique and the simplicity of implementation, aluminum is finally chosen as the material of the cantilever beam, whose specific parameters are shown in Table 1.

## 4. Results

### 4.1. Effect of Threshold Voltage Change

**Figure 5:**
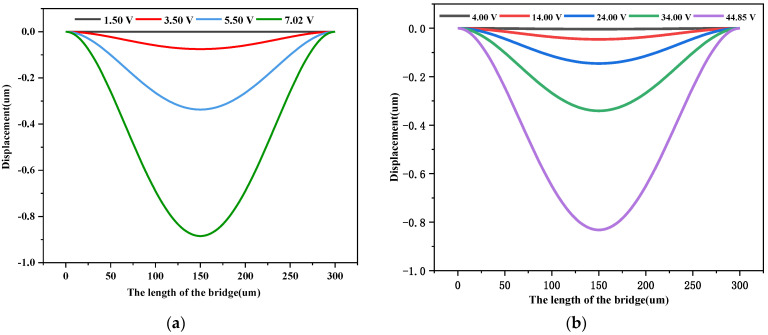

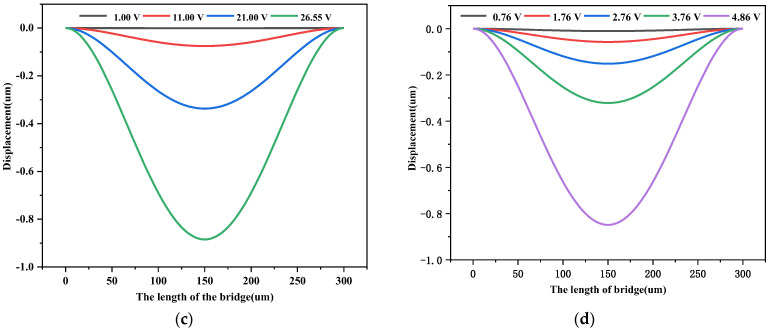
The relationship curves between the deflection of the switch and the control voltage filled with different materials: (**a**) the surrounding dielectric is air; (**b**) the surrounding dielectric is glycerol; (**c**) the surrounding dielectric is silicone oil; (**d**) the surrounding dielectric is water.

Analysis shows that the cantilever drops only when the applied control voltage reaches the threshold voltage. The pull-in phenomenon occurs when the drive voltage is high enough and the initial distance d of the signal line between the cantilevers dropped to d0 = 2d/3d. Figure 5 shows the voltage displacement curves of the RF MEMS switch filled with different media.

From Figure 5, it can be seen that the threshold voltage of the RF MEMS switch is different when air, glycerin, silicone oil, and water are filled into the switch, namely 44.85 V, 7.02 V, 26.55 V, and 4.86 V, respectively. After the four media shown in the figure are filled into the switch, the threshold voltage of the switch changes in the same direction. When the applied control voltage causes the switch to drop one-third of its height, the cantilever bar has a large deflection in the range of a small voltage as the control voltage continues to increase. The dielectric constant of the filling medium is 37 for glycerol, 2.6 for silicone oil, and 78.36 for water. The calculation shows that the threshold voltage decreases to 1/ε as the dielectric constant of the environment increases. This suggests that the threshold voltage of the RF MEMS switch can be effectively reduced by encapsulating a high dielectric constant insulating liquid.

### 4.2. Effect of Change of Switch Filling Material on Response Time

**Figure 6:**
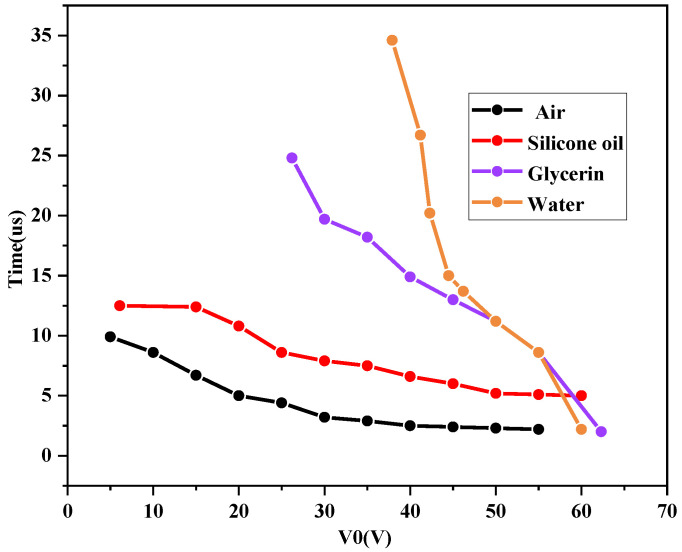
Curves of response time and driving voltage of different material filled switches.

The study of the fall time of the switch shows that different filler media have different threshold voltages, and the cantilever beam starts to fall only when the drive voltage reaches a certain value. In addition, the fall time of the switch decreases as the drive voltage increases. Figure 6 shows the curves of response time and drive voltage. It can be seen from Figure 6 that the response time of the switch decreases with the increase of the drive voltage when four dielectrics are used as the filling material. In addition, the response time of the switch decreased rapidly near the threshold voltage and decreased slowly as the threshold voltage increases. Therefore, the trigger voltage slightly above the threshold voltage should be selected to ensure normal triggering of the RF MEMS switch and to reduce the response time of the switched RF MEMS. However, a high drive voltage increases the shock rate of the cantilever and the dielectric charging effect, shortening the lifetime of the switch. Therefore, setting the drive voltage at one and a half to two times the threshold voltage is a more reasonable approach. The drive voltage is about 19.5 V with glycerol as the filler material of the switch, 35.2 V with silicone oil as the filler material, 7.1 V with water as the filler material, and 44.1 V with air as the filler material. Experimental results show that the response time of the RF MEMS switches filled with different media is less than 46.2 μs.

The experimental results show that the response times of the RF MEMS switch filled with different materials were different. The response time of glycerol was 12.5 µs, that of silicone oil was 24.8 µs, that of water was 9.9 µs, and that of air was 34.6 µs. The results show that although the threshold voltage was reduced by filling with insulating liquid with high a dielectric constant such as glycerol and water, the viscosity coefficient of glycerol at room temperature was 1.65 B.N-S/m^2^. As the driving voltage increases, the response time decreases to some extent, but from the slope of the response times in Figure 6, the response time decreases fastest for the switch with air as the filling medium, but the threshold voltage is higher. When the trigger voltage is 20 V, the response time of the switch is about 9.8 s, while the unfilled RF MEMS switch does reach the threshold voltage and the switch is in the off state. When the drive voltage was increased above 60 V, the switching time of the unfilled medium was shorter than the switching times of glycerol, silicone oil, and other substances.

### 4.3. Effect of Change of Switch Filling Material on Impact Velocity

**Figure 7:**
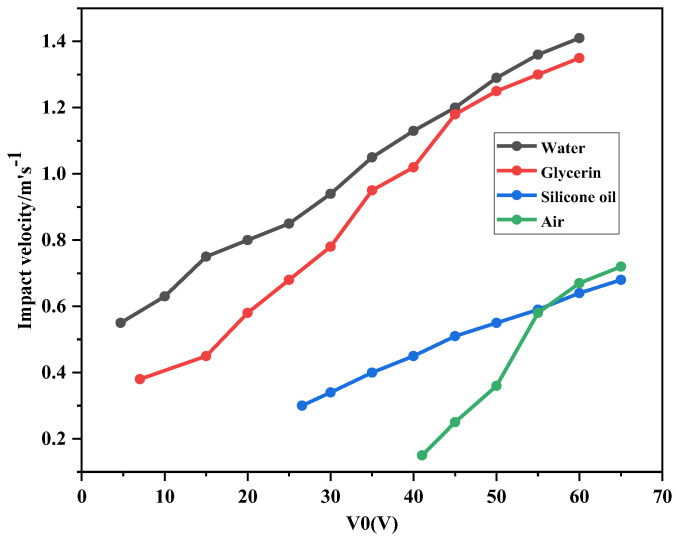
Curves of relation between impact velocity and driving voltage of different material filled switches.

As the control voltage increased, the rate of descent of the switch increases. When the drive voltage reaches the threshold voltage, the falling speed of the switch increases more slowly. As can be seen from the velocity-voltage curve in Figure 7, when the control voltage is 0 to 30 V, the impact velocity is between 0.35 and 0.9 m/s, and the unfilled switch has not yet reached the threshold voltage. At a control voltage of 7.02 V, the impact velocity of glycerol is 0.41 m/s. At a control voltage of 41.04 V, the impact velocities of the filling media silicone oil, water, glycerol, and air are 1.15 m/s, 0.21 m/s, 0.79 m/s, and 0.21 m/s, respectively. It can be observed that the variation of the viscosity coefficient and the driving voltage has a considerable influence on the impact velocity of the switch.

Therefore, when different insulating fluids are used to reduce the threshold voltage of RF MEMS switches, the viscosity coefficients of the insulating fluids need to be compared to control the response time and impact velocity, which can improve the service life of the switches.

### 4.4. Effect of Change of Switch Filling Material on Capacitance

When the drive voltage is applied to the cantilever, the cantilever is subjected to an electrostatic force close to the dielectric layer, the distance between the cantilever and the dielectric layer decreases, and the capacitance increases. When the cantilever is in close contact with the dielectric layer, the capacitance of the switch reaches the maximum. The experimental data for such problems are shown in Table 2: C_L_ is the theoretical capacitance of the switch in the off state, Cs is the actual capacitance of the switch in the off state, C is the actual capacitance of the switch in the open state, and ratio is the capacitance ratio of the switch.

**Figure 8:**
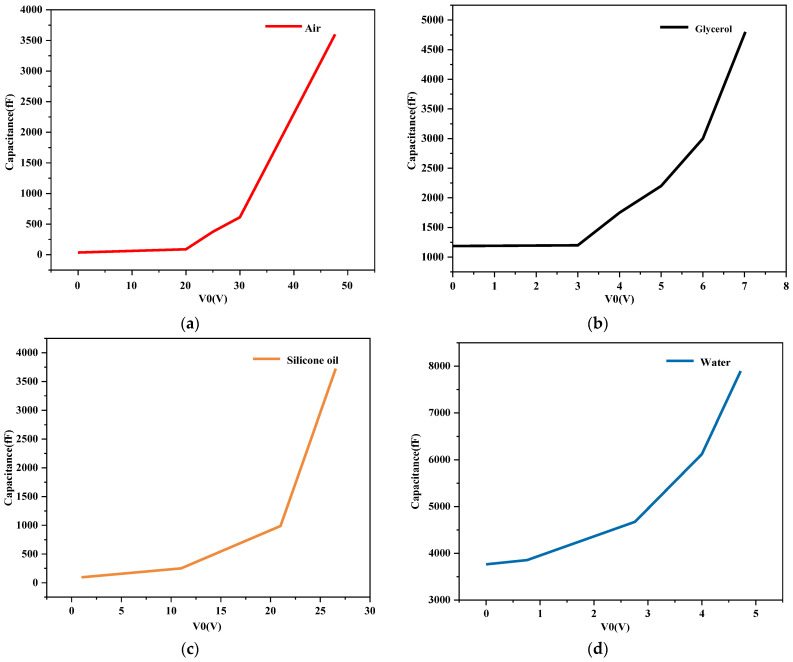
Changes in switch capacitance after RF MEMS switch was filled with different materials: (**a**) air; (**b**) glycerol; (**c**) silicone; (**d**) water.

When the control voltage is applied to the RF MEMS switch, the switching capacitance varies with the deflection of the cantilever, as shown in Figure 8. The ratio of the switching capacitances affects the characteristics of the switched RF MEMS. From Table 2 it can be seen that the maximum capacitance ratio of the RF MEMS switch with air as the filling dielectric is 75.9. Since the capacitance switching ratio of the RF MEMS switch was larger than 15, the performance of the RF MEMS switch could be guaranteed, while the capacitance ratio of the RF MEMS switch with glycerol was 4.0, which affects the characteristics of the switch.

In summary, although liquids with high dielectric constants such as glycerol and water can effectively lower the threshold voltage of RF MEMS switches, the capacitance rate of the RF MEMS switch was decreased when the permittivity was high, which affected the switching characteristics of the device. The experimental results show that the relative tolerance of the insulating liquid is less than 9.8, which can make the capacitive switching ratio of the RF MEMS switch larger than 15, thereby ensuring the performance of the RF MEMS switch. Thus, silicone oil was ultimately selected as the filling material of the switch.

### 4.5. Effect of Change of Switch Filling Material on Insertion Loss and Isolation

**Figure 9:**
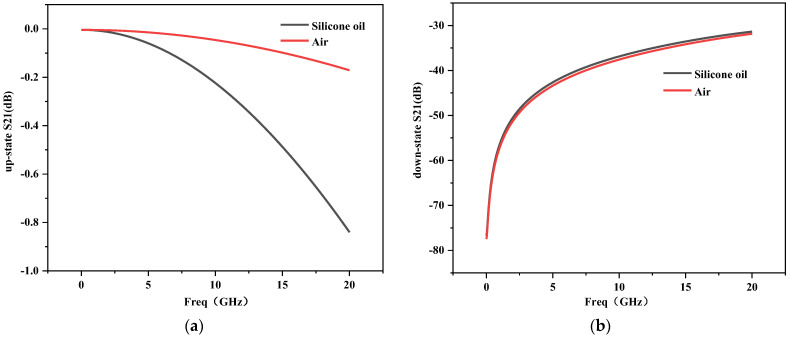
(**a**) Insertion loss when the switch is in the open state; (**b**) isolation when the switch is in the down state.

The insertion loss of this working switch is very good due to the small up-state capacitance of the cantilever beam. Although the insertion loss of the silicone oil-filled switch is slightly higher than that of the air-encapsulated switch, the insertion loss of the switch increases to 0.84 dB when the frequency rises to 20 GHz, which is in a very good range. Ideally, the smaller the isolation of the switch when the cantilever beam is in the down state, the better, i.e., the larger the |S21| is, the better. From Figure 9b we can see that the isolation of the switch after filling with silicone oil is better than that of the air-packed RF MEMS switch.

## 5. Conclusions

In this study, an RF MEMS switch filled with a liquid medium is proposed and designed, and compared with an air-filled RF MEMS switch. The choice of the filling medium is particularly important for the switch. Although the high dielectric constant dielectric filling of the switch can effectively reduce the threshold voltage of the switch, the high dielectric constant dielectric filling of the switch has a greater effect on the capacitance ratio of the switch. The viscosity coefficient of the filler liquid also affects the shock speed of the switch at the same voltage. After pre-screening of various substances, glycerin and silicone oil were selected as the main comparison objects. Since the dielectric constant of silicone oil is relatively low, and response time loss is relatively low, based on the results of simulation experiments, silicone oil is selected as the filling medium for the final switch developed in this paper.

The results show that RF MEMS switches encapsulated with glycerin, silicone oil, water, and other liquids can reduce the threshold voltage and board shock strength, and avoid the negative effects of external conditions such as ambient humidity on RF MEMS switch performance. However, when RF MEMS switches are filled with an insulating liquid dielectric with high dielectric constant, which leads to a lower switching capacitance ratio and affects the switching performance to some extent. After comparative analysis, silicone oil was finally adopted as the encapsulation material for the liquid dielectric. The results show that the threshold voltage is 26.55 V after filling with silicone oil, which is 43% lower under the same air-encapsulated switching conditions. When the trigger voltage is 30.02 V, the response time is 10.12 μs, and the impact speed is only 0.35 m/s. The frequency is good in 0–20 GHz switching performance, and the insertion loss is up to 0.84 dB. Meanwhile, it is found that the relative dielectric constant of the insulating liquid is less than 9.8, which can make the capacitive switching ratio of the RF MEMS switch increase to more than 15, thus ensuring the switch performance of the RF MEMS switch has reference significance to the fabrication of RF MEMS switches.

## Figures and Tables

**Table 1:** Parameters of the cantilever beam.

Materials	Youngs Modulus	Poisson Ratio	Density	Length	Width	Height
Al	69 GPa	0.35	2.7 g/cm^3^	300 μm	60 μm	2 μm

**Table 2:** Capacitance ratio of switches with different filler materials.

	Air	Glycerol	Water	Silicone Oil
C_L_(fF)	37.2	1653	3868	83.2
Cs(fF)	2794	4720	7897	3568
C(fF)	36.8	1180	3652	78.9
ratio	75.9	4.0	2.1	42.9

## Data Availability

The data used to support the findings of this study are available from the corresponding author upon request.
